# Studyholism and Attachment Style: A Study among Italian University Students

**DOI:** 10.3390/bs14100865

**Published:** 2024-09-25

**Authors:** Yura Loscalzo, Marco Giannini

**Affiliations:** Department of Health Sciences, School of Psychology, University of Florence, 50135 Florence, Italy; marco.giannini@unifi.it

**Keywords:** studyholism, study addiction, workaholism, work addiction, study engagement, OCD, obsession

## Abstract

Studyholism (or obsession with studying) is a new potential clinical condition introduced in the literature in 2017. Since then, growing research has supported its conceptualization as a clinical disorder and highlighted some potential intervention targets, namely trait worry, social anxiety, negative interpretation bias, and defense mechanisms. The present study aims to extend the literature concerning psychodynamic-related constructs that might constitute targets for interventions aimed at reducing Studyholism by investigating the role of attachment in 1073 students (*M*_age_ = 23.48 ± 3.77), balanced concerning civil status (i.e., currently being single or involved in a relationship/non-single). Among the main findings, we found that insecure attachment—mainly preoccupied attachment—is a positive predictor of Studyholism in both non-single and single students. However, there are also some differences depending on the civil status. Finally, (single) disengaged studyholics have a statistically significant lower level of secure attachment than (single) engaged studyholics. In conclusion, this study showed the value of distinguishing between non-single and single students when investigating the role of attachment. Regarding problematic overstudying specifically, the study provided support for its definition as a clinical disorder, also with evidence of the appropriateness of its OCD-related conceptualization. Finally, it suggests preoccupied (insecure) attachment as a target to reduce Studyholism by fostering in students the feeling of being loved and deserving of being loved in their current adult relationships.

## 1. Introduction

Studyholism (or obsession toward study) is a new potential clinical condition introduced in the literature in 2017 [1]. In brief, as highlighted by Loscalzo’s [2] recent narrative review—Studyholism research [3,4,5,6,7,8] supported its definition as an OCD-related disorder characterized by high levels of study-related obsessive-compulsive symptoms, which might also be associated with high study engagement levels. In fact, Loscalzo and Giannini [1] suggested distinguishing between two studyholic types: engaged and disengaged studyholics. Even if they differ concerning study engagement levels and areas of impairment, they are both clinical forms of Studyholism (in contrast with the first theorization by Loscalzo and Giannini [1]) [3,4]. In the literature about problematic overstudying, there is also a research group [9] that defined it in terms of a behavioral addiction (i.e., study addiction)—namely as being characterized by the seven core components of addictions: salience, tolerance, mood modification, relapse, withdrawal, conflict, and problems [10]—and that investigated it by adopting this theoretical framework, e.g., [11,12,13,14].

Therefore, considering that the research on problematic overstudying is still in its infancy, it is crucial to analyze this new potential clinical condition further to gain insight into its proper definition as an OCD-related (or, more generally, internalizing) disorder or behavioral addiction (or, externalizing disorder). Also, it is vital to analyze the predictive role of clinical constructs, which might help in understanding if problematic overstudying might be conceptualized as a clinical disorder and, more importantly, shedding light on potential targets of preventive and clinical interventions. Additionally, the literature consistently showed that engaged and disengaged studyholics have different relationships with some antecedents and outcomes, while they do not differ on some variables, such as trait worry or sensation seeking [3,4,6,8]. For college students, it has been shown that disengaged studyholics are more impaired in the academic and affective areas, while they are less impaired in the social area [3]. Moreover, disengaged studyholics have higher levels of obsessive-compulsive symptoms than engaged studyholics [6]. Regarding antecedents, Loscalzo and Giannini [3] found that disengaged studyholics have lower perfectionistic strivings (the positive component of perfectionism) and higher perfectionistic concerns (the negative facet of perfectionism) than engaged studyholics. About study-related perfectionism, it is higher in engaged studyholics [3]. Therefore, it is critical to tailor preventive and clinical interventions to the studyholic type and, consequently, to analyze potential predictors/interventions’ targets by distinguishing between the two studyholic types. 

In this vein, previous literature highlighted that trait worry is a strong predictor of Studyholism in both adolescents and youths, suggesting that programs already tested for reducing trait worry might be applied to reduce Studyholism or, if applied in the school context, to prevent it [3,4]. Another study, which focused on the role of social anxiety in adolescents, pinpointed the value of screening highly socially anxious adolescents for the likely presence of high Studyholism, as well as the potential of social anxiety treatments for prevention and clinical interventions [8]. In addition, with specific reference to the results of studyholics’ interpretation style in social and non-social situations, Loscalzo and Giannini [8] proposed that clinical interventions should focus on non-social situations (especially school-related performance and situations) to decrease the tendency to interpret these situations negatively or neutrally (while increasing their positive interpretation).

While the findings from the studies mentioned above [3,4,8] might be framed in the cognitive-behavioral therapies approach, there are also findings supporting the value of psychodynamic therapies (e.g., Freudian therapy, Jungian therapy, and Sandplay Therapy) for reducing Studyholism levels [2]. More specifically, Loscalzo and Giannini [7] recently pointed out the role of defense mechanisms as predictors of Studyholism. Defense mechanisms are one of the core contributions of psychoanalysis [15,16]. They are used by everybody in everyday situations—as they are activated by threatening or anxiety-provoking situations—to handle stress and negative emotions [17]. Therefore, defense mechanisms might be defined as pathological when used excessively or when the person uses immature/maladaptive defenses [18,19]. In brief, regarding the stronger predictors of Studyholism, it is positively predicted by the following maladaptive defenses: regression, projective identification, help-rejecting complaining, withdrawal, and somatization. Moreover, it is negatively predicted by omnipotence (an image-distortion defense style). Finally, concerning adaptive defenses, suppression is a negative predictor, while task orientation and pseudo-altruism are positive predictors [7]. 

Another core construct in the psychodynamic area is attachment, whose importance has been increasingly recognized in other approaches, including cognitive psychology and systematic approaches [20]. John Bowlby introduced the construct of attachment, which Mary Ainsworth then enriched and enlarged, in contrast with both behavioral and Freudian perspectives. He elaborated on the psychoanalytic concepts by integrating findings from other areas, such as etiology, evolutionistic theories, and cognitive psychology [21]. In brief, Bowlby avoided an interpretation focused only on the individual and his/her internal states. Instead, he focused on the relationship between the child and the caregiver (not necessarily the mother), which he conceptualized as having a biological/innate ground. Therefore, in contrast with the Freudian theory that foresees a developmental path (distinguished in phases) that is the same across different children, Bowlby suggested that, at the beginning of life, the person has the potential to develop different evolutive paths. A specific path manifests due to the relationship between the child and the caregiver [21,22]. The different outcomes of the early child–caregiver relationships have been categorized by Mary Ainsworth—based on the Strange Situation procedure (that evaluates the child’s reaction to the separation and reunion with the mother)—into four main categories: secure, insecure-avoidant, insecure-ambivalent, and disorganized [21,23,24,25]. 

It is crucial to emphasize that Bowlby [26,27,28] highlighted the enduring nature of the attachment need, which spans the entire life and influences future intimate relationships. This continuity is attributed to the development of the internal working models of attachment. Based on the child’s perception of the caregiver’s responsiveness and lovability, these models originated in childhood, but they continue to shape expectations in significant relationships throughout life [21,29,30,31]. Moreover, the literature showed that attachment—besides influencing adult intimate relationships—plays a vital role in other life domains, such as students’ adjustment and developmental advances during the college years [32]. For example, in the 1990s, Rice et al. [33] showed that security of attachment is positively associated with various indexes of students’ adjustment, including academic and emotional facets. Then, Lapsley and Edgerton [34] reported that secure adult attachment is positively associated with college adjustment (in contrast with fearful and preoccupied attachment). More recently, Wright and Lyon [35] highlighted that attachment influences social provision areas in the early phases of college, which in turn might favor college students’ persistence. In this field of research, scholars also analyzed the role of mediating and moderating variables in the relationship between attachment and college students’ adjustment, including problem copying styles and healthy levels of separation-individuation [36,37].

Evaluating attachment style in adults is challenging, considering that neither Bowlby nor Ainsworth provided indications about conceptualizing and measuring it after childhood [38]. George et al. [39] and Hazan and Shaver [40] have made the first attempts. George et al. [39] designed the Adult Attachment Interview, which analyzes the adults’ descriptions of their childhood experiences with attachment figures. Hazan and Shaver [40] created instead a categorical forced-choice self-report measure based on adult romantic relationships, classifying adults as being characterized by secure, anxious-ambivalent, or anxious-avoidant attachment. Later, scholars developed other self-report scales, including the Experiences in Close Relationships (ECR) scale [41], which was later revised (ECR-R; [42]). Brennan et al. [41] integrated the scales available to measure adult attachment; then, they demonstrated that adult romantic attachment might be organized through a bi-dimensional factor structure: anxiety (i.e., fear of abandonment and hyper-vigilance about rejection) and avoidance (i.e., feeling uncomfortable with closeness and avoiding intimacy with others). Recently, Giannini et al. [20] also designed a scale (included in the broader Psychological Treatment Inventory—PTI [43]) that assesses via Likert-scale self-report items the four (categorial) attachment styles: secure, ambivalent, avoidant, and unresolved (or disorganized).

### The Present Study 

Analyzing the attachment style in Studyholism is of critical importance from a clinical perspective, as insecure attachment is a risk factor across different diagnoses—such as depression, anxiety, OCD, eating disorders, and addictive behaviors—and from mild distress to severe personality disorders, and even schizophrenia [44,45,46,47,48,49,50,51,52,53]. Therefore, it is possible to speculate that insecure attachment might also play a role in Studyholism. 

Considering the two divergent perspectives on problematic overstudying—that is, Studyholism [1] and Study Addiction [9]—van Leeuwen et al. [54] reported that the results from the studies about attachment in OCD are mixed and complex to compare. However, their meta-analysis showed an association of medium to large effect size between attachment anxiety and OCD, as well as an association of medium effect size between attachment avoidance and OCD [54]. Though, they also concluded by underscoring that attachment anxiety and attachment avoidance might show different relations with the various OCD dimensions, hence suggesting that future studies assess how different OCD subtypes relate to specific attachment styles [54]. In line with this, Pozza et al. [55] stressed that the literature highlighted that insecure attachment characterizes OCD but that studies are not convergent concerning the specific form of attachment involved, with some studies also suggesting that only attachment anxiety is associated with OCD severity [56,57]. As the ECR [41,42] dimensions do not allow a categorical differentiation of attachment insecurity, Pozza et al. [55] used the Attachment Style Questionnaire [58], which measures confidence in relationship (a feature of secure attachment) and the following facets of insecure attachment: need for approval and preoccupation with relationships (for anxiety attachment), and discomfort with closeness and relationships as secondary to achievement (for avoidant attachment). Their study showed that the need for approval is the most important predictor of an OCD diagnosis; although, most importantly, they found evidence that different OCD symptoms might be related to different facets of attachment insecurity. Therefore, they suggested that the heterogeneous nature of OCD might partially explain the contradictory results in OCD literature.

About the addiction literature, it is worthwhile noting that some scholars argued that addictive behaviors might be conceptualized as an attachment disorder since the use of drugs or the involvement in rewarding (non-substance) behaviors might serve the role of compensating for the lack of intimacy and giving the feeling of having a secure base [59,60,61,62]. Focusing on gambling—which is the only officially recognized behavioral addiction in the DSM-5 [63]—research suggested insecure attachment (both anxious and avoidant) as a characteristic of gamblers [64,65,66]. The recent meta-analysis by Ghinassi and Casale [67] highlighted that—with some exceptions—insecure attachment is a vulnerability factor for gambling. Regarding attachment types, they reported consistent results concerning the association between dismissive (or avoidant) attachment and gambling, while preoccupied attachment was not associated with gambling (despite a few exceptions). The weaker evidence was about the disorganized (or unresolved) attachment, with some studies not finding an association and others highlighting a positive relationship. Therefore, Ghinassi and Casale [67] speculated that different types of insecure attachments lead to different games gamblers use to satisfy their specific attachment needs. Similarly, Macía et al. [68] found that predictive variables might change based on the specific type of addiction: for alcohol and drug addiction, attachment has a crucial role; for gambling and compulsive spending (behavioral) addiction, gambling motives play a greater role than attachment. 

In conclusion, it is possible to argue that insecure attachment plays a role in both OCD and gambling/behavioral addictions and that the literature supports the need to analyze attachment with a more detailed view (hence, also looking for the different attachment styles) as there might be attachment-related differences based on the specific type/subtype of disorder, both in the OCD and behavioral addiction area. Therefore, the present study will not shed light on the internalizing and/or externalizing nature of problematic overstudying, given that there is not a sharp difference in attachment style between OCD and addiction disorders. However, analyzing the predictive value of (insecure) attachment will illuminate the appropriateness of conceptualizing Studyholism as a clinical disorder. Also, gathering insights for Studyholism treatment and prevention will be possible based on the specific attachment features that will arise.

Hence, this study aims to analyze the attachment style characterizing Studyholism (in its three components: obsessions, compulsions, and social impairment [69]) using both a dimensional and categorial approach. Therefore, we aim to explore (i) which attachment dimensions (i.e., anxiety and avoidance attachment) and attachment styles (i.e., secure, preoccupied, avoidant, and unresolved) predict Studyholism and Study Engagement; (ii) if there are differences in attachment between students characterized by high/low levels of Studyholism/Study Engagement (which might correspond to a clinical and control group for Studyholism); (iii) if there are differences in attachment between engaged and disengaged studyholics. Since—to the best of the authors’ knowledge—this is the first research about attachment style in problematic overstudying and study engagement, and given the lack of specific attachment types in different clinical diagnoses, *we cannot posit specific hypotheses*. However, based on previous Studyholism and attachment literature—e.g., [6,7,54,67]—we expect Studyholism to be characterized by insecure attachment and study engagement by secure attachment. 

In our study, we included study engagement (which is a positive attitude toward study) and analyzed Studyholism in its three facets in line with previous research that showed the importance of controlling for study engagement when analyzing Studyholism, as well as of differentiating between engaged and disengaged studyholics and between the three Studyholism components as they might have different relationships with the same variables, e.g., [1,3,7,69,70,71].

## 2. Materials and Methods

### 2.1. Participants

We gathered 1073 Italian college students aged between 18 and 56 (*M*_age_ = 23.48 ± 3.77; 75.8% females), balanced concerning civil status, that is, being currently involved (i.e., engaged, married or co-habitant) or not (i.e., single or widow) in a relationship. More specifically, 596 students are currently engaged (55.5%), and 477 students are not (44.5%). Table 1 shows the descriptive statistics for the total sample and the two sub-groups, which we labeled non-single and single students. 

### 2.2. Materials 

#### 2.2.1. Studyholism Inventory (SI-10) [72,73]

The Studyholism Inventory is a 10-item self-report instrument that evaluates Studyholism and Study Engagement. Each scale comprises four items (plus a filler item). A sample item for the Studyholism scale is “I cannot relax because of worries about studying,” while for Study Engagement, it is “My desire to get good grades motivates me to study.” The items are preceded by a head sheet with questions concerning study habits (e.g., studying on the weekend and time spent studying). The response format is a 5-point Likert scale ranging between 1 (Strongly Disagree) and 5 (Strongly Agree). We administered the Italian version by Loscalzo and Giannini [73], which has good psychometric properties. More specifically, regarding internal reliability, Cronbach’s alpha value is 0.84 for Studyholism and 0.81 for Study Engagement [73]. 

#### 2.2.2. Studyholism Inventory—Extended Version (SI-15) [69]

The SI-15 is a 15-item self-report instrument that measures Studyholism in its three components: Obsessions (e.g., “I cannot relax because of worries about studying”), Compulsions (e.g., “I study much more than what is necessary”), and Social Impairment (e.g., “My family and/or my friends complain because I study too much”). Each scale comprises five items. The Obsessions scale contains the four items of the SI-10 Studyholism scale plus an additional item. The response format is a 5-point scale ranging between 1 (Strongly Disagree) to 5 (Strongly Agree). We administered the Italian version by Loscalzo and Giannini [69]. The Cronbach’s alpha values for Obsessions, Compulsions, and Social Impairment subscales are, respectively, 0.85, 0.82, and 0.86 [69]. 

#### 2.2.3. Psychological Treatment Inventory—Attachment Styles Scale (PTI-ASS) [20]

The Psychological Treatment Inventory (PTI) [43] is a comprehensive instrument comprising two forms. There is a patient, or self-report, version that addresses the following macro-areas: validity, resources, clinical area/symptomatology, and psychological treatment (including the attachment styles scale; PTI-ASS [20]). Moreover, there is a clinical version, a form filled out by the clinician that covers relational modalities and evaluation of the treatment’s outcome. 

In this study, we administered the PTI-ASS only, which allows for evaluating the attachment styles through 22 items based on a 5-Likert scale ranging between 1 (Not at all) and 5 (A great deal). The PTI-ASS assesses four attachment styles: secure, ambivalent, avoidant, and unresolved. The current study participants received the (original) Italian version, which proved to have good psychometric properties [20]. The Cronbach’s alpha values for the four subscales are: Secure = 0.80, Preoccupied = 0.81, Avoidant = 0.75, and Unresolved = 0.80. The instructions of the PTI-ASS ask the person to fill out the questionnaire by thinking about the partner (the most important partner—present or past) or, in the case of no previous relationships, to think about a strong friendship. Regarding sample items for the four scales, an example of secure attachment is being able to build satisfying relationships, while for preoccupied attachment is being afraid of being abandoned in intimate relationships. For avoidant attachment, an example is the belief that it is better to live alone than with a partner. Finally, for unresolved attachment, an example is being mistreated by the partner.

#### 2.2.4. Experiences in Close Relationships—Revised (ECR-R) [42]

This is a 36-item self-report scale that evaluates attachment style through two scales: Attachment Anxiety (e.g., being afraid of not being really loved by the partner) and Attachment Avoidance (e.g., not feeling comfortable when the partner wants intimacy). The response format is a 7-point Likert scale ranging between 1 (Strongly disagree) and 7 (Strongly agree). In the current study, we administered the Italian version by Busonera et al. [74]. Regarding the instructions, the ECR-R asks about relationships in general, not with specific reference to a present partner. However, it specifies that “partner” should not be meant for a friend or family member; also, the terms “close” and “intimate” should be meant for psychological/emotional intimacy and not only physical/sexual intimacy. Cronbach’s α is 0.90 and 0.89 for the Anxiety and Avoidance scale, respectively. 

### 2.3. Procedure

First, we obtained research approval from the Ethical Committee of the University of Florence. Then, we created an online questionnaire containing a first section asking about demographic data (e.g., gender and age), the SI-10, the SI-15, the attachment scale from the PTI, and the ERC-R. Since the questionnaire was completed online, we reported the information required by the informed consent on the first page. We required participants to check the box stating that they agreed to participate in the research by filling out the questionnaire on the subsequent pages (or to deny and close the questionnaire if they did not want to participate). We recruited participants by spreading the research invitation throughout social networks, aiming to reach participants outside Tuscany and across different areas of study. 

### 2.4. Data Analysis 

We performed the analyses using SPSS.26 (IBM, Chicago, IL, USA) and AMOS.20 (IBM, Chicago, IL, USA).

Preliminarily, we calculated the descriptive statistics of all the study variables, including their skewness and kurtosis. Then, we normalized one of the PTI scales (i.e., unresolved attachment) by removing the outliers and then using linear interpolation to replace the missing values we created. Then, as an additional preliminary step—given that the attachment questionnaires we administered asked participants to refer to a significant relationship—we performed ANOVAs to evaluate if students, engaged (non-single students) or not in a relationship (single students), differ on the PTI-ASS and ERC-R scales to decide if the path analysis model should have been tested on the total sample or by differentiating the two groups. Based on the ANOVAs results, we ran a path analysis model on the two groups separately, with the PTI-ASS and ERC-R scales as predictors of the SI-15 scales and the SI-10 Study Engagement scale. We used the cut-off values provided by Byrne [75], Hu and Bentler [76], and Reeve et al. [77] to evaluate the fit of the two models.

Finally, we evaluated if there are attachment differences between students scoring high and low on the SI-10 Studyholism and Study Engagement scales (through MANOVAs) and between engaged and disengaged studyholics (using Mann–Whitney tests) differentiating between students currently or not in a relationship. We referred to the SI-10 cut-off values [73] for high/low Studyholism and Study Engagement and the two studyholic types.

## 3. Results

### 3.1. Preliminary Analyses

First, given that the analyses of this study require the variables to be normally distributed, we calculated the descriptive statistics of all the study variables, including their skewness and kurtosis. Appendix A shows the results of these analyses, also distinguishing between non-single (*n* = 596) and single (*n* = 477) participants.

Next, as a second preliminary step, given that the attachment questionnaires we administered asked participants to refer to a significant relationship, we performed ANOVAs to evaluate if non-single students (*n* = 596) and single students (*n* = 477) differ on the PTI-ASS and ERC-R scales, aiming to evaluate if the path analysis model and group-differences analyses planned for this research should have been performed on the total sample (*n* = 1073) or separately for the two sub-groups of participants (i.e., non-single and single students). As shown in Appendix B, the two sub-groups differ on all the attachment variables. Therefore, the analyses have been performed separately for the two sub-groups. 

### 3.2. Path Analysis 

First, we calculated the zero-order correlations between the variables included in the path analysis models: SI-15 scales, PTI-ASS scales, ERC-C scales, and SI-10 Study Engagement scale. As shown in Table 2, in the non-single group, all the SI-15 scales correlate negatively with PTI-ASS secure attachment and positively with the other PTI-ASS and ECR-R scales (except for the lack of correlation with PTI-ASS avoidant attachment). The single group reports a similar pattern of correlations, even if there is an additional lack of significant correlation with the PTI-ASS unresolved attachment. Regarding Study Engagement, there are few statistically significant correlations (for the non-single group only): it correlates positively with PTI-ASS secure attachment and negatively with the ERC-C Attachment Avoidance scale. Therefore, these results provide preliminary evidence for an association between Studyholism and negative attachment style (in both the sub-groups) and between Study Engagement and positive attachment style in the non-single group.

Then, we ran two path analysis models (for non-single and single students separately) with the three SI-15 subscales and the SI-10 Study Engagement scale as outcomes of the PTI-ASS and ERC-R scales. The model showed an excellent fit to the data for both the non-single [CFI = 1.00; GFI = 1.00; RMSEA = 0.000(CI 90% 0.000–0.086); SRMR = 0.0043] and the single group [CFI = 0.999; GFI = 0.999; RMSEA = 0.057(CI 90% 0.000–0.149); SRMR = 0.0144]. However, the attachment scales especially explain SI-15 Obsessions compared to the other dependent variables. The percentage of variance explained for the variables—for the non-single and single groups, respectively—are Obsessions = 13.5% and 18.7%; Compulsions = 5.6% and 4.4%; Social Impairment = 5.4% and 5.3%; Study Engagement = 2.8% and 2.0%. There are few statistically significant predictors. In the non-single group, the highest *β* value is for Attachment Anxiety on Obsessions, while in the single group, it is for Preoccupied Attachment on Obsessions. Study Engagement is predicted only by Secure Attachment (in the non-single group only). The SI-15 Compulsions and Social Impairment scales are not predicted by the attachment variables (for both groups). Table 3 shows the path analysis models’ standardized path weights (and *p* values).

### 3.3. Differences in Attachment between Students with High and Low Levels of Studyholism and Study Engagement 

To analyze if there are differences between students scoring high and low on the SI-10 subscales, we performed MANOVAs with high and low levels of Studyholism/Study Engagement as the independent variables and the PTI-ASS and ERC-R scales as dependent variables (see Table 4 for the descriptives of these analyses). Students have been grouped based on their high/low levels of Studyholism/Study Engagement using the cut-off scores by Loscalzo and Giannini [73]. MANOVAs have been conducted separately for non-single students and single students.

In the non-single group, there are 100 students with high Studyholism (16.8%) and 80 with low Studyholism (13.4%); regarding Study Engagement, 94 students have a high score (15.8%), and 68 have a low score (11.4%). The MANOVA with the PTI-ASS scales as dependent variables highlighted a multivariate statistically significant effect of Studyholism on the PTI-ASS scales: *F*(4,157) = 10.53, *p* < 0.001, η^2^ = 0.19. More specifically, follow-up ANOVAs showed that students with high Studyholism score lower on secure attachment and higher on preoccupied and unresolved attachment styles (see Table 4). The MANOVA with Study Engagement instead highlighted the lack of a multivariate statistically significant effect on the PTI-ASS scales: *F*(4,157) = 2.24, *p* = 0.067, η^2^ = 0.05.

Concerning the MANOVAs with the ERC-R scales as dependent variables, we found again a multivariate statistically significant effect for Studyholism, *F*(2,177) = 18.70, *p* < 0.001, η^2^ = 0.17, but not for Study Engagement, *F*(2,159) = 2.30, *p* = 0.103, η^2^ = 0.03. Then, follow-up ANOVAs showed that students with high Studyholism score higher on the Attachment Anxiety scale (see Table 4).

In the single group, there are 99 students with high Studyholism (20.8%) and 62 with low Studyholism (13.0%); regarding Study Engagement, 64 students have a high score (13.4%), and 69 have a low score (14.5%). 

The MANOVA with the PTI-ASS scales as dependent variables highlighted a multivariate statistically significant effect of Studyholism on the PTI-ASS scales: *F*(4,156) = 15.81, *p* < 0.001, η^2^ = 0.29. More specifically, follow-up ANOVAs showed that students with high Studyholism score lower on secure attachment and higher on preoccupied attachment (see Table 5). The MANOVA with Study Engagement instead highlighted the lack of a multivariate statistically significant effect on the PTI-ASS scales: *F*(4,128) = 1.02, *p* = 0.400, η^2^ = 0.03.

Concerning the MANOVAs with the ERC-R scales as dependent variables, we found, again, a multivariate statistically significant effect for Studyholism, *F*(2,158) = 24.96, *p* < 0.001, η^2^ = 0.24; but not for Study Engagement, *F*(2,130) = 1.18, *p* = 0.311, η^2^ = 0.02. Then, follow-up ANOVAs showed that students with high Studyholism score higher on both the Attachment Anxiety and Attachment Avoidance scales (see Table 5). 

### 3.4. Differences in Attachment between Engaged and Disengaged Studyholics

Using Loscalzo and Giannini’s [73] cut-off values, we screened the participants (non-single group), and we found these percentages: Engaged Student, *n* = 7, 1.2%; Engaged Studyholic, *n* = 27, 4.5%; Detached Student, *n* = 12, 2.0%; Disengaged Studyholic, *n* = 11, 1.8%. Then, we performed Mann–Whitney tests to evaluate if there are differences between the two types of studyholic. As shown by Table 6, there are no differences between the two studyholic types in the non-single group (or students in a relationship).

Regarding single students, we found these percentages: Engaged Student, *n* = 7, 1.5%; Engaged Studyholic, *n* = 19, 4.0%; Detached Student, *n* = 15, 3.1%; Disengaged Studyholic, *n* = 8, 1.7%. Then, Mann–Whitney tests showed a statistically significant difference in secure attachment only, with Disengaged Studyholics scoring lower than Engaged Studyholics (*r* = −0.49). Table 7 shows the results of these analyses.

## 4. Discussion

This study analyzed the attachment style characterizing Studyholism, a new potential clinical condition related to problematic overstudying, in its three components: obsessions, compulsions, and social impairment, using both a dimensional and categorical approach. More specifically, it evaluated attachment through two measures: the ECR-R [42], which allows evaluating attachment anxiety and attachment avoidance (two dimensions indicating insecure attachment generally), and the PTI-ASS [20], which allows measuring the four types of attachment style: secure, preoccupied, avoidant, and unresolved.

Before performing the main analyses—given that the PTI-ASS and the ECR-R are filled by participants referring to a significant relationship—we evaluated if there were differences in the attachment variables between non-single and single students. Since we found statistically significant differences in all the attachment variables, we performed the statistical analyses separately for the two groups. However, it is interesting to note that these preliminary results align with the attachment theory that emphasizes the enduring nature of the attachment need, which—through internal working models—influences future intimate relationships [26,27,28]. In fact, students characterized by higher levels of secure attachment and lower levels of insecure attachment are also involved in a relationship (compared to single students).

The zero-order correlations ran as a preliminary step for the path analysis model(s) with the attachment scales as predictors of both Studyholism (in its three facets) and study engagement provided initial support for the insecure attachment style (in both the sub-groups) in Studyholism and for the secure attachment style in study engagement (even if only in the non-single group). Then, the path analysis model(s), which allows controlling for the effect of the variables included in the model, showed that Obsessions is the Studyholism component whose variance is explained the most by the attachment variables (compared to Compulsions and Social Impairment from the SI-15 [69]). Moreover, the other two Studyholism facets are not statistically significantly predicted by attachment. Regarding predictors, the highest *β* value is for ECR-R Anxiety Attachment (*β* = 0.19) in the non-single group, while it is for PTI-ASS Preoccupied Attachment (*β* = 0.26) in the single group. These two scales address similar facets of insecure attachment, namely the tendency to fear being abandoned by the partner. The higher *β* value in the single group—as well as the higher percentage (about 5% more) of Obsessions variance explained by attachment in the single group—might be due to the role played by internal working models [26,27,28]. Insecure attachment, as being related to the feeling of not being loved and not deserving to be loved [21], might reduce the capacity to be in a stable relationship; therefore, its role might be more evident in studyholics who have not been able to overcome the insecurity experienced with the caregiver and then build a stable relationship in which they are currently involved.

Interestingly, our results underscore that it is the obsessive component that is critical in defining problematic overstudying as a clinical disorder, as (insecure) attachment is associated with clinical disorders [44,45,46,47,48,49,50,51,52,53]. Therefore—considering that compulsion is a transdiagnostic factor across disorders, including addictions [78], while obsessions are a feature of OCD [63]—we suggest that the path analysis results support the OCD-related framework [1] against the behavioral addiction conceptualization [9]. These findings also align with a previous study that showed that the obsessive component is the critical variable in predicting higher psychopathology and worse academic performance (compared to compulsions and functional impairment) [6]. 

It is important to note that the statistically significant predictors of the SI-15 Obsessions scale are somewhat different in the two sub-groups. In both the sub-groups, preoccupied attachment (from the PTI-ASS) and attachment anxiety (from the ECR-R) are positive predictors of Studyholism. These two scales assess similar features of preoccupied attachment, such as the fear of abandonment or of not being loved as much as the person does. This type of attachment might be present in both sub-groups of students in line with the fact that it might be evident either in a person currently in a relationship (in the form of the fear of being abandoned by the current partner) or in single students who might be single due to their difficulties in feeling safe in a relationship. Instead, unresolved attachment is a positive predictor in the non-single group only (*β* = 0.11), while avoidant attachment is a positive predictor in the single group (*β* = 0.11). We speculate this difference is because unresolved attachment—as being related to violent relationships—might be seen with a higher likelihood in those students currently involved with a partner. Single students are not involved with a sentimental partner; therefore, they are unlikely to report this type of attachment. Also, the PTI-ASS foresees the possibility for single participants to refer to the past most significant partner or (if they never had a partner) to a present significant friendship. Therefore, it is unlikely that they will select a violent relationship as the significant one to fill the PTI-ASS, even if they have experienced it in the past. On the other hand, avoidant attachment—concerning the tendency to avoid stable relationships and intimacy—might be more prevalent in single students since their current civil status might suggest they are actively avoiding a sentimental partner, which is in line with the attachment style feature of being afraid of intimacy. 

Even if the literature is inconsistent about the attachment style typical of OCD and behavioral addictions, it is interesting to note that our results further support the OCD-related conceptualization [1]. Pozza et al. [55] found that the need for approval—which is similar to preoccupied attachment—is the most significant predictor of OCD; like in our study, PTI-ASS preoccupied attachment and ECR-R attachment anxiety are the (two) variables that predict the most the SI-15 Obsessions scale in both non-single and single students. Moreover, Ghinassi and Casale [67] showed in their meta-analysis that preoccupied attachment style (with a few exceptions) is not associated with gambling. 

Finally, study engagement is the variable predicted at a lower extent in both sub-groups, indicating that attachment plays a greater role in explaining Studyholism (or a negative attitude toward studying) than study engagement (or a positive attitude toward studying). Also, it is predicted (positively) by secure attachment in the non-single group only. The lack of prediction for the single group might be explained by the fact that these students, not being involved in an intimate relationship, might be characterized by a secure attachment at a lower extent than students in a relationship. In sum, these results align with the notion that attachment is a crucial variable for clinical disorders, so it is not a strong predictor for study engagement as it is not a clinical disorder but rather represents a positive attitude toward studying. Thus, this result highlights that Studyholism, even if related to a study behavior like study engagement, might be conceptualized as a clinical disorder despite being related to a common behavior [79]. However, to avoid fostering a negative and deterministic view of attachment, it is important to underline that the literature showed that attachment plays a critical role in explaining psychopathology [44,45,46,47,48,49,50,51,52,53] but also in students’ adjustment [32,33,34,35,36,37].

We also conducted MANOVAs to analyze if students with high Studyholism/Study Engagement differ from students with low levels of Studyholism/Study Engagement regarding attachment. Compared to path analysis, and specifically with regards to Studyholism, here we might have a comparison between a “clinical” (high studyholics) and a “control” (low/no studyholics) group, as Studyholism and study engagement are independent variables rather than the outcome variables. The results showed no differences between high/low levels of study engagement on attachment scales, neither in non-single nor single students, further supporting the role of attachment in clinical disorders. In line with this, regarding high Studyholism, we found instead that both non-single and single groups scored lower on PTI-ASS secure attachment and higher on PTI-ASS preoccupied attachment and ECR-R attachment anxiety compared to low Studyholism. Moreover, in the non-single sub-group, studyholics also score higher on PTI-ASS unresolved attachment, while single students also score higher on ECR-R attachment avoidance (which is similar to PTI-ASS avoidant attachment, even if there is no statistically significant difference for this variable). Therefore, MANOVAs further supported civil-status differences concerning attachment and, more generally, the proper conceptualization of Studyholism as a clinical disorder, given that it further pointed out that insecure attachment levels differ between clinical and control groups.

Finally, in line with the critical value of distinguishing between the two studyholic types for preventive and clinical purposes [1,3,4], we used Mann–Whitney tests to evaluate if there are attachment differences between engaged and disengaged studyholics. While we did not find differences in the group of non-single students, a difference arose among single students: (single) disengaged studyholics have lower levels of secure attachment than (single) engaged studyholics. Therefore, in line with the previous results that showed attachment-related differences based on students’ civil status, we find a difference between the two studyholic types only for single students. Also, we provided further support to distinguish between the two studyholic types when considering different areas of impairment. As previously reported, disengaged studyholics show a higher impairment in the psychological, physical, and academic areas but are less impaired in the social area (where engaged studyholics experience higher impairment) [3,4,6,8]. The present study also shows that (single) disengaged studyholics, besides being characterized by insecure attachment like engaged studyholics, also have lower levels of secure attachment, thus being more affected in their ability to build good intimate relationships with a partner or another significant person and providing further evidence about the differences between engaged and disengaged studyholics concerning some antecedents. 

## 5. Conclusions

This study is the first addressing attachment style in problematic overstudying by adopting a comprehensive approach analyzing Studyholism in its three components (obsessions, compulsions, and social impairment) and its two subtypes (engaged and disengaged Studyholism) while controlling for the effect of study engagement (as a positive attitude toward study that might be present in some studyholics [1]). Also, we analyzed attachment both dimensionally (attachment anxiety and avoidance) and through its four types (secure, preoccupied, avoidant, unresolved).

Regarding the limitations of the present study, we used self-report measures, and the participants are mainly females living in Central Italy. Also, there is a higher percentage of psychology students and a few first-year and fourth-year students. However, the sample is broad and comprises a range of majors across various Italian regions. Future studies could analyze if there are age-related differences by gathering more students in the “older” age range (e.g., older than 30) or across different academic years. Moreover, qualitative interviews or focus groups could be implemented to gain a more in-depth insight into problematic overstudying, both generally and concerning its relationship with attachment. Finally, it is essential to extend Studyholism research across non-Western and non-individualistic countries to analyze if there are culture-related differences. In this vein, the (psychological) literature about Studyholism might benefit from the insights that might come through an interdisciplinary analysis of the construct, for example, from sociology and philosophy.

There are several strengths of this paper. Concerning attachment research generally, our results about civil-status differences highlighted the critical need to differentiate the analyses—regardless of the clinical condition under investigation—based on the civil status of the person, as there are convergences but also differences in attachment between people currently involved in a relationship (or, non-single students) and single students. Regarding problematic overstudying specifically, we gathered insights from theoretical and clinical perspectives. 

From the theoretical side, we found support for the definition of Studyholism as a clinical disorder, given that insecure attachment arose as a positive predictor in both non-single and single students, which is in line with previous literature highlighting attachment as a transdiagnostic factor across clinical disorders [44,45,46,47,48,49,50,51,52,53]. Moreover, even if this should be taken cautiously, given the lack of specific attachment types found in different clinical disorders, the path analysis model(s) findings seem to support the OCD-related/internalizing framework [1] against the addiction/externalizing framework [9], as indicated by obsessions being the only Studyholism facet predicted by attachment and by the comparison of our results regarding the attachment type that arose with previous OCD and gambling literature [16,55]. Finally, we provided further support for distinguishing between engaged and disengaged studyholics when analyzing Studyholism features. 

From a clinical perspective, our findings have significant implications. The role played by preoccupied attachment style in studyholics, especially in single students, supports the use of psychodynamic therapies as a useful treatment for reducing Studyholism, as previously suggested by a study concerning defense mechanisms [7]. Furthermore, considering the civil status and studyholic type differences that emerged, it is critical to tailor preventive and clinical interventions to the studyholic type and the civil status of the student. Given the role of preoccupied attachment, clinical interventions should foster the feeling of being loved and deserving of being loved in current (adult) relationships.

## Figures and Tables

**Table 1:** Descriptive statistics of the demographic and study-related variables on the total sample (*n* = 1073) and by the two sub-groups: non-single students (*n* = 596) and single students (*n* = 477).

Variable	Variable	Non-Single Group	Single Group	Total Sample
Gender	Males	21.6%	27.5%	24.2%
	Females	78.4%	72.5%	75.8%
Age	Range	18–48	18–56	18–56
	M(SD)	23.90(3.78)	22.94(3.69)	23.48(3.77)
Residence in Italy	North	26.7%	27.9%	27.2%
	Center	55.0%	53.0%	54.1%
	South	17.8%	18.9%	18.3%
Civil Status	Cohabitant	12.2%	-	6.8%
	Engaged	86.4%	-	48.0%
	Married	1.3%	-	44.4%
	Widow/er	-	0.2%	0.7%
	Single	-	99.8%	0.1%
Working Status	Only student	66.3%	74.6%	70.0%
	Also worker	33.7%	25.4%	30.0%
Area of Study	Psychology	38.3%	30.6%	34.9%
	Engineering	12.1%	14.3%	13.0%
	Medical Studies	6.7%	6.5%	6.6%
	Economy	8.1%	8.6%	8.3%
	Languages	3.2%	3.4%	3.3%
	Biology	5.2%	7.1%	6.1%
	Architecture	4.9%	5.7%	5.2%
Year of study	1	9.7%	15.7%	12.4%
	2	13.4%	17.4%	15.2%
	3	25.2%	26.6%	25.8%
	4	9.1%	10.9%	9.9%
	5	39.8%	27.9%	34.5%
	6 °	1.0%	1.0%	1.0%
Repeated a school year	Yes	9.7%	6.9%	8.5%
	No	90.3%	93.1%	91.5%
Being out of course at university	Yes	14.8%	16.4%	15.5%
	No	85.2%	83.6%	84.5%
Studying on the weekend	Yes	85.1%	89.3%	87.0%
	No	14.9%	10.7%	13.0%
Hours a day of study	Range	0–12	0–10	0–12
	M(SD)	4.16(1.98)	4.02(1.82)	4.10(1.91)
Days a week of study	Range	0–7	1–7	0–7
	M(SD)	5.07(1.29)	5.06(1.28)	5.07(1.28)
Hours a day of study before exams	Range	0–18	0–12	0–18
	M(SD)	6.58(2.35)	6.19(1.05)	6.45(2.27)
Grade Point Average *	Range	20–31	18–31	18–31
	M(SD)	27.08(1.98)	26.63(2.11)	26.88(2.05)

Note. There are a few missing cases for some variables; ° only for medical students; * = Italian sufficient grades are between 18 and 30, 31 is used here to indicate the addition of “laude”.

**Table 2:** Zero-order correlations of the study variables by non-single students (*n* = 596) and single students (*n* = 477).

	1	2	3	4	5	6	7	8	9	10
1.Obs	-									
2.Comp	0.50 *** 0.44 ***	-								
3.SocImp	0.44 *** 0.46 ***	0.66 *** 0.72 ***	-							
4.StEng	0.20 *** 0.18 ***	0.42 *** 0.37 ***	0.27 *** 0.32 ***	-						
5.Secure	−0.14 *** −0.14 **	−0.18 *** −0.13 **	−0.17 *** −0.15 ***	0.17 *** 0.09	-					
6.Preocc	0.31 *** 0.38 ***	0.16 *** 0.15 ***	0.13 ** 0.17 ***	−0.04 *0.06*	−0.26 *** −0.11 *	-				
7.Avoid	0.04 *0.09*	0.05 *0.05*	0.05 *0.05*	−0.02 −*0.01*	−0.22 *** −0.19 ***	−0.09 * −0.11 *	-			
8.Unres ^#^	0.25 *** 0.07	0.16 *** 0.06	0.17 *** 0.04	−0.05 *−0.07*	−0.29 *** −0.12 **	0.35 *** 0.12 **	0.06 *0.04*	-		
9.AttAnx	0.34 *** 0.36 ***	0.20 *** 0.16 ***	0.19 *** 0.16 ***	−0.05 *0.03*	−0.38 *** −0.15 ***	0.75 *** 0.74 ***	0.02 −0.07	0.46 *** 0.23 ***	-	
10.AttAv	0.12 ** 0.17 ***	0.16 *** 0.13 **	0.17 *** 0.15 ***	−0.12 ** −0.09	−0.71 *** −0.63 ***	0.19 *** 0.13 **	0.34 *** 0.31 ***	0.28 *** 0.13 **	0.38 *** 0.17 ***	-

Note. *** *p* ≤ 0.001; ** *p* ≤ 0.01; * *p* < 0.05; values in italics refer to the group of single students. # = variable normalized, missing values replaced using linear interpolation. Obs = Obsessions; Comp = Compulsions; SocImp = Social Impairment; StEng = Study Engagement; Secure = Secure Attachment; Preocc = Preoccupied Attachment; Avoid = Avoidant Attachment; Unres = Unresolved Attachment; AttAnx = Attachment Anxiety; AttAv = Attachment Avoidance.

**Table 3:** Standardized path weights for the path analysis models.

Dependent Variable	Predictor	Group	*β*	*p*
Obsessions	Secure Attachment	Non-single	−0.03	n.s.
		Single	−0.02	n.s.
	Preoccupied Attachment	Non-single	0.14	0.021
		Single	0.26	<0.001
	Avoidant Attachment	Non-single	0.05	n.s.
		Single	0.11	0.017
	Unresolved Attachment	Non-single	0.11	0.008
		Single	−0.02	n.s.
	Attachment Anxiety	Non-single	0.19	0.003
		Single	0.17	0.009
	Attachment Avoidance	Non-single	−0.05	n.s.
		Single	0.06	n.s.
Compulsions	Secure Attachment	Non-single	−0.09	n.s.
		Single	−0.07	n.s.
	Preoccupied Attachment	Non-single	0.04	n.s.
		Single	0.08	n.s.
	Avoidant Attachment	Non-single	0.02	n.s.
		Single	0.03	n.s.
	Unresolved Attachment	Non-single	0.07	n.s.
		Single	0.02	n.s.
	Attachment Anxiety	Non-single	0.09	n.s.
		Single	0.08	n.s.
	Attachment Avoidance	Non-single	0.03	n.s.
		Single	0.05	n.s.
Social Impairment	Secure Attachment	Non-single	−0.05	n.s.
		Single	−0.08	n.s.
	Preoccupied Attachment	Non-single	−0.002	n.s.
		Single	0.13	n.s.
	Avoidant Attachment	Non-single	0.01	n.s.
		Single	0.03	n.s.
	Unresolved Attachment	Non-single	0.09	n.s.
		Single	−0.01	n.s.
	Attachment Anxiety	Non-single	0.10	n.s.
		Single	0.04	n.s.
	Attachment Avoidance	Non-single	0.07	n.s.
		Single	0.07	n.s.
Study Engagement	Secure Attachment	Non-single	0.16	0.007
		Single	0.05	n.s.
	Preoccupied Attachment	Non-single	−0.03	n.s.
		Single	0.09	n.s.
	Avoidant Attachment	Non-single	0.03	n.s.
		Single	0.03	n.s.
	Unresolved Attachment	Non-single	−0.01	n.s.
		Single	−0.06	n.s.
	Attachment Anxiety	Non-single	0.05	n.s.
		Single	−0.01	n.s.
	Attachment Avoidance	Non-single	−0.03	n.s.
		Single	−0.07	n.s.

**Table 4:** Follow-up ANOVAs. PTI-ASS and ERC-R scales by low and high Studyholism (SH) and Study Engagement (SE). Non-single group.

Variable		Level	*n*	*M(SD)*	*F ^§^*	*p*	*Partial η^2^*
PTI Secure	SH	Low	80	21.84(2.68)	3.96	0.048	0.02
		High	100	20.93(3.30)			
		Total	180	21.33(3.06)			
	SE	Low	68	20.22(3.16)	-	-	-
		High	94	21.65(3.33)			
		Total	162	21.05(3.32)			
PTI Preoccupied	SH	Low	80	11.69(4.17)	32.32	<0.001	0.15
		High	100	15.92(5.51)			
		Total	180	14.04(5.38)			
	SE	Low	68	14.12(5.28)	-	-	-
		High	94	13.73(5.10)			
		Total	162	13.89(5.16)			
PTI Avoidant	SH	Low	80	12.92(3.71)	0.85	n.s.	0.005
		High	100	12.37(4.23)			
		Total	180	12.62(4.01)			
	SE	Low	68	13.10(4.16)	-	-	-
		High	94	13.04(5.02)			
		Total	162	13.07(4.66)			
PTI Unresolved	SH	Low	80	6.21(0.61)	24.34	<0.001	0.12
		High	100	7.02(1.37)			
		Total	180	6.66(1.17)			
	SE	Low	68	6.87(1.23)	-	-	-
		High	94	6.48(1.10)			
		Total	162	6.64(1.17)			
ERC-R Attachment Anxiety	SH	Low	80	43.42(14.07)	37.52	<0.001	0.17
		High	100	60.92(22.22)			
		Total	180	53.14(20.89)			
	SE	Low	68	56.04(20.99)	-	-	-
		High	94	52.58(19.04)			
		Total	162	54.04(19.89)			
ERC-R Attachment Avoidance	SH	Low	80	35.94(13.53)	1.65	n.s.	0.009
		High	100	38.91(16.80)			
		Total	180	37.59(15.46)			
	SE	Low	68	42.84(16.60)	-	-	-
		High	94	37.01(18.01)			
		Total	162	39.46(17.62)			

Note. For Study Engagement, follow-up ANOVAs are not reported since the multivariate test is not statistically significant. ^§^ = for Studyholism, df = 1,178. PTI = PTI-ASS, Psychological Treatment Inventory, Attachment Styles Scale; ECR-R = Experiences in Close Relationships—Revised.

**Table 5:** Follow-up ANOVAs. PTI-ASS and ERC-R scales by low and high Studyholism (SH) and Study Engagement (SE). Single group.

Variable		Level	*n*	*M(SD)*	*F ^§^*	*p*	*Partial η* ^2^
PTI Secure	SH	Low	62	18.42(3.89)	8.69	0.004	0.05
		High	99	16.32(4.67)			
		Total	161	17.13(4.50)			
	SE	Low	69	16.81(4.08)	-	-	-
		High	64	17.62(4.38)			
		Total	133	17.20(4.23)			
PTI Preoccupied	SH	Low	62	12.61(4.76)	57.39	<0.001	0.27
		High	99	18.80(5.21)			
		Total	161	16.42(5.86)			
	SE	Low	69	15.09(5.33)	-	-	-
		High	64	16.59(5.92)			
		Total	133	15.81(5.65)			
PTI Avoidant	SH	Low	62	16.44(4.88)	0.35	n.s.	0.002
		High	99	16.94(5.50)			
		Total	161	16.74(5.26)			
	SE	Low	69	16.46(5.79)	-	-	-
		High	64	16.05(5.17)			
		Total	133	16.26(5.48)			
PTI Unresolved	SH	Low	62	7.01(1.94)	1.48	n.s.	0.01
		High	99	7.41(2.10)			
		Total	161	7.25(2.04)			
	SE	Low	69	7.15(1.64)	-	-	-
		High	64	7.08(2.04)			
		Total	133	7.12(1.84)			
ERC-R Attachment Anxiety	SH	Low	62	55.61(17.05)	47.01	<0.001	0.23
		High	99	77.66(21.41)			
		Total	161	69.17(22.52)			
	SE	Low	69	65.97(21.50)	-	-	-
		High	64	68.55(24.54)			
		Total	133	67.21(22.96)			
ERC-R Attachment Avoidance	SH	Low	62	52.35(20.82)	7.20	0.008	0.04
		High	99	62.01(23.04)			
		Total	161	58.29(22.64)			
	SE	Low	69	59.65(21.71)	-	-	-
		High	64	54.66(21.37)			
		Total	133	57.25(21.61)			

Note. For Study Engagement, follow-up ANOVAs are not reported since the multivariate test is not statistically significant. ^§^ = for Studyholism, df = 1,159. SI-10 = PTI = PTI-ASS, Psychological Treatment Inventory, Attachment Styles Scale; ECR-R = Experiences in Close Relationships—Revised.

**Table 6:** Mann–Whitney tests for attachment scales by studyholic type. Non-single group.

Attachment Scale	*U*	*Z*	*p*	Type of Student	*Median*	*n*
PTI Secure	96.00	−1.70	ns	Disengaged Studyholic	18.00	11
				Engaged Studyholic	21.00	27
PTI Preoccupied	99.00	−1.60	ns	Disengaged Studyholic	21.00	11
				Engaged Studyholic	18.00	27
PTI Avoidant	118.50	−0.97	ns	Disengaged Studyholic	10.00	11
				Engaged Studyholic	12.00	27
PTI Unresolved	90.00	−2.00	ns	Disengaged Studyholic	8.00	11
				Engaged Studyholic	6.00	27
ERC-R Anxiety	116.00	−1.05	ns	Disengaged Studyholic	84.00	11
				Engaged Studyholic	58.00	27
ECR-R Avoidance	146.50	−0.06	ns	Disengaged Studyholic	45.00	11
				Engaged Studyholic	42.00	27

Note. PTI = PTI-ASS, Psychological Treatment Inventory, Attachment Styles Scale; ECR-R = Experiences in Close Relationships—Revised.

**Table 7:** Mann–Whitney tests for attachment scales by studyholic type. Single group.

Attachment Scale	*U*	*Z*	*p*	Type of Student	*Median*	*n*
PTI Secure	28.00	−2.56	0.009	Disengaged Studyholic	13.50	8
				Engaged Studyholic	19.00	19
PTI Preoccupied	72.50	−0.19	ns	Disengaged Studyholic	20.50	8
				Engaged Studyholic	20.00	19
PTI Avoidant	53.00	−1.23	ns	Disengaged Studyholic	17.50	8
				Engaged Studyholic	14.00	19
PTI Unresolved	59.50	−0.95	ns	Disengaged Studyholic	7.50	8
				Engaged Studyholic	6.00	19
ERC-R Anxiety	64.50	−0.61	ns	Disengaged Studyholic	92.50	8
				Engaged Studyholic	92.00	19
ECR-R Avoidance	58.50	−0.93	ns	Disengaged Studyholic	65.00	8
				Engaged Studyholic	57.00	19

Note. PTI = PTI-ASS, Psychological Treatment Inventory, Attachment Styles Scale; ECR-R = Experiences in Close Relationships—Revised.

## Data Availability

Data might be required, for research purposes only, by writing to the corresponding author.

## Appendix A. Normality Assessment of the Study Variables

As shown in Table A1, except for one PTI variable (Unresolved Attachment), all the variables are normally distributed, as indicated by their skewness and kurtosis values between −1 and +1. However, based on the content of the PTI Unresolved Attachment (addressing abusive relationships), a skewed and kurtotic distribution was expected, particularly for participants currently involved in a relationship (or, non-single group).

To include the PTI Unresolved Attachment scale in the analyses, we normalized its distribution by removing the outliers. Specifically, to preserve the data as much as possible, we referred to Bentler’s [80] cut-off criterion of 5 to indicate a variable as not violating the normal distribution. We normalized the variable on the total sample (*n* = 1073) by removing 22 participants (those who scored higher than 15 on the scale). More specifically, we created a missing value for ten students from the non-single group and 12 participants from the single group. However, distinguishing between non-single (*n* = 596) and single students (*n* = 477), the kurtosis value was still higher than the cut-off of five for the non-single group (skewness = 2.79, kurtosis = 8.70). Therefore, to avoid removing (unnecessary) data from the single group, we repeated the process of removing the outliers in the non-single group only, resulting in the deletion of an additional 17 participants (who scored higher than 11). Finally, we used the missing case substitution function (linear interpolation) to replace the missing values created to normalize the variable (i.e., 12 substitutions for the single group and 27 substitutions for the non-single group).

**Table A1:** Descriptive statistics of the variables on the total sample (*n* = 1073) and by the two sub-groups: non-single students (*n* = 596) and single students (*n* = 477).

Variable	Group	M(SD)	Skewness	Kurtosis
SI-10 Studyholism	Total	14.50(3.94)	−0.51	−0.47
	Non-single	14.52(3.84)	−0.49	−0.50
	Single	14.47(4.06)	−0.53	−0.45
SI-10 Study Engagement	Total	14.50(3.46)	−0.43	−0.23
	Non-single	14.71(3.44)	−0.44	−0.22
	Single	14.24(3.49)	−0.43	−0.24
SI-15 Obsessions	Total	16.74(5.27)	−0.30	−0.83
	Non-single	16.70(5.17)	−0.31	−0.81
	Single	16.80(5.41)	−0.30	−0.85
SI-15 Compulsions	Total	10.82(4.68)	0.66	−0.20
	Non-single	10.68(4.68)	0.69	−0.11
	Single	10.99(4.68)	0.63	−0.29
SI-15 Social Impairment	Total	9.52(4.51)	1.04	0.53
	Non-single	9.42(4.47)	1.12	0.80
	Single	9.65(4.57)	0.96	0.24
PTI Secure Attachment	Total	19.26(4.15)	−0.69	0.12
	Non-single	21.19(3.06)	−0.76	0.17
	Single	16.85(4.08)	−0.39	−0.11
PTI Preoccupied Attachment	Total	14.96(5.06)	0.15	−0.86
	Non-single	13.91(4.76)	0.32	−0.65
	Single	16.26(5.13)	−0.12	−0.89
PTI Avoidant Attachment	Total	14.76(4.97)	0.52	−0.04
	Non-single	13.28(4.24)	0.52	−0.06
	Single	16.62(5.19)	0.31	−0.27
PTI Unresolved Attachment	Total	7.20(2.44)	3.23	12.88
	Non-single	6.95(2.24)	4.10	21.40
	Single	7.52(2.64)	2.52	7.32
PTI Unresolved Attachment_Nor *	Total	6.96(1.74)	2.29	5.18
	Non-single	6.56(1.05)	2.10	3.98
	Single	7.23(1.96)	1.85	2.87
ECR-R Attachment Anxiety	Total	60.16(20.49)	0.35	−0.56
	Non-single	53.32(18.53)	0.68	0.02
	Single	68.71(19.60)	0.01	−0.45
ECR-R Attachment Avoidance	Total	47.84(20.18)	0.58	−0.29
	Non-single	39.18(15.90)	0.92	0.68
	Single	58.66(19.75)	0.16	−0.43

Note. Nor = normalized; * = missing values replaced using linear interpolation. SI-10 = Studyholism Inventory; SI-15 = Studyholism Inventory, Extended Version; PTI = PTI-ASS, Psychological Treatment Inventory, Attachment Styles Scale; ECR-R = Experiences in Close Relationships—Revised.

## Appendix B. Differences between Non-Single and Single Students Concerning the Study Variables

Table A2 shows that the two sub-groups differ in all the attachment variables, with non-single students being characterized by higher levels of secure attachment (PTI-ASS), lower levels of preoccupied, avoidant, and unresolved attachment (PTI-ASS), as well as by lower levels of attachment anxiety and attachment avoidance (ECR-R).

Finally, we also analyzed if the two sub-groups differ on the SI-10 and SI-15: the ANOVAs (see Table A2) only highlighted a difference in Study Engagement (with non-single students scoring higher than single students).

Table A1 shows the descriptive statistics for all the variables analyzed through ANOVAs.

**Table A2:** Follow-up ANOVAs by the two sub-groups: non-single (*n* = 596) and single students (*n* = 477).

Variable	F °	*p*	*Partial η* ^2^
PTI Secure Attachment	395.37	<0.001	0.27
PTI Preoccupied Attachment	60.13	<0.001	0.05
PTI Avoidant Attachment	135.14	<0.001	0.11
PTI Unresolved Attachment_Normalized #	21.89	<0.001	0.02
ECR-R Attachment Anxiety	173.72	<0.001	0.14
ECR-R Attachment Avoidance	320.47	<0.001	0.23
SI-10 Studyholism	0.04	n.s.	0.000
SI-10 Study Engagement	4.78	0.029	0.004
SI-15 Obsessions	0.09	n.s.	0.000
SI-15 Compulsions	1.12	n.s.	0.001
SI-15 Social Impairment	0.73	n.s.	0.001

Note. # = missing values replaced using linear interpolation; ° = df 1,1071. SI-10 = Studyholism Inventory; SI-15 = Studyholism Inventory, Extended Version; PTI = PTI-ASS, Psychological Treatment Inventory, Attachment Styles Scale; ECR-R = Experiences in Close Relationships—Revised.
